# Rapid and Sensitive Detection of Inactivated SARS-CoV-2 Virus via Fiber-Optic and Electrochemical Impedance Spectroscopy Based Aptasensors

**DOI:** 10.3390/bios14050231

**Published:** 2024-05-07

**Authors:** Can Xiao, Nan Wang, Yuechao Zhao, Xuemei Liu, Hui Li, Aixue Huang, Lin Wang, Xinhui Lou, Bo Gao, Ningsheng Shao

**Affiliations:** 1 Beijing Institute of Basic Medical Sciences, Beijing 100850, China; xiaocan@bmi.ac.cn (C.X.); zhaoyuechao@bmi.ac.cn (Y.Z.); liuxuemei@bmi.ac.cn (X.L.); lihui1@bmi.ac.cn (H.L.); huangaixue@bmi.ac.cn (A.H.); wanglin1@bmi.ac.cn (L.W.); 2 Department of Chemistry, Capital Normal University, Xisanhuan North Road. 105, Beijing 100048, China; 2210702032@cnu.edu.cn (N.W.); xinhuilou@cnu.edu.cn (X.L.)

**Keywords:** SELEX, aptamer, inactivated SARS-CoV-2, fiber-optic aptasensor, electrochemical impedance aptasensor

## Abstract

The development of rapid detection tools for viruses is vital for the prevention of pandemics and biothreats. Aptamers that target inactivated viruses are attractive for sensors due to their improved biosafety. Here, we evaluated a DNA aptamer (named as 6.9) that specifically binds to the inactivated SARS-CoV-2 virus with a low dissociation constant (K_D_ = 9.6 nM) for the first time. Based on aptamer 6.9, we developed a fiber-optic evanescent wave (FOEW) biosensor. Inactivated SARS-CoV-2 and the Cy5.5-tagged short complementary strand competitively bound with the aptamer immobilized on the surface of the sensor. The detection of the inactivated SARS-CoV-2 virus was realized within six minutes with a limit of detection (LOD, S/N = 3) of 740 fg/mL. We also developed an electrochemical impedance aptasensor which exhibited an LOD of 5.1 fg/mL and high specificity. We further demonstrated that the LODs of the FOEW and electrochemical impedance aptasensors were, respectively, more than 1000 and 100,000 times lower than those of commercial colloidal gold test strips. We foresee that the facile aptamer isolation process and sensor design can be easily extended for the detection of other inactivated viruses.

## 1. Introduction

Severe acute respiratory syndrome coronavirus 2 (SARS-CoV-2) caused the emergence and worldwide spread of coronavirus disease 2019 (COVID-19). The development of rapid and accurate on-site detection of SARS-CoV-2 is still vital for the prevention of the current and future pandemics.

Currently, there are various virus detection methods, including real-time reverse transcription polymerase chain reaction (RT-PCR) and antibody-based detection methods [1]. RT-PCR, which is the gold standard for the detection of the SARS-CoV-2 infection, has played an important role in transmission control [2]. However, highly skilled personnel and a critically sterile manipulating environment are necessary for RT-PCR detection, which hampers its applications, especially in point-of-care testing (POCT). In addition, its other drawbacks, including the time consumption for viral processing, biosafety issues, and the occurrence of false negative and/or false positive results [3,4], are also notable. As an essential alternative or supplementary means for the detection of SARS-CoV-2 infection, many POCT methods have been developed, among which lateral flow immunoassays (LFIAs) are the most widely used technique, which is attributed to their simple manipulation and fast response [5]. Nevertheless, considering that commercially available antigen tests based on LFIAs for SARS-CoV-2 are effective with a high viral load sample (cycle threshold (Ct) ≤ 25 in RT-PCR), insensitivity is the most undeniable drawback of LFIAs [6]. Thus, biosensors with high sensitivity and rapid response are still in demand for viral infection screening.

Aptamers are single-stranded DNA or RNA oligonucleotides that are isolated with a screening technique named “systematic evolution of ligands by exponential enrichment” (SELEX) [7,8]. They can recognize various targets, including ions [9], small molecules [10], proteins [11], and even tissue slides [12,13], by forming specific conformations. Aptamers have become the central sensing elements in diverse types of biosensors in recent years [14,15]. As the biorecognition element in biosensors, aptamers exhibit unique advantages over antibodies, such as ease of synthesis, thermal stability, and batch-to-batch reproducibility [16]. Aptamers have also been used for SARS-CoV-2 detection. Previously investigated aptamers were mainly against the spike glycoprotein [17,18,19,20,21,22,23] and nucleocapsid protein [24,25,26,27,28,29] of SARS-CoV-2, and they were further developed into aptasensor-based detection techniques based on electrochemical [17,24], colorimetric [18,19,25], and fluorescent strategies [20,26]. Aptamers that target inactivated viruses are attractive because of the feasibility of avoiding biosafety issues and the convenience of their operation [30]. Unfortunately, so far, there are no reports on the isolation of aptamers that target inactivated SARS-CoV-2 viruses.

Magnetic cross-linking precipitation (MCP)–SELEX is a facile solution-based variant of SELEX in which the target is incubated with a library of aptamers in solution, followed by the capture of the bound aptamers via the highly efficient chemical cross-linking between amino groups of targets and the activated carboxylic acid groups on magnetic beads [31]. The possible interference of a solid surface for target immobilization is avoided, leading to a high library enrichment efficiency. MCP–SELEX was initially used for the isolation of protein-binding aptamers. Recently, it has been used for the efficient isolation of small molecule-binding aptamers with high specificity and affinity [32]. There have hitherto been no reports on the application of MCP–SELEX to the synthesis of inactivated virus-binding aptamers.

Fiber-optic evanescent wave (FOEW) sensors are based on the fluorescence excitation of a fluorophore within an evanescent field. A fluorophore, which is typically labeled on the recognition ligand or target, is attached to the surface of optical fibers. FOEW sensors offer advantages such as low cost, ease of operation, automated data collection, a small size, and easy maintenance [32,33]. FOEW aptasensors have been analyzed for the sensitive detection of various types of targets, including heavy metal ions [34], proteins [33], and exosomes [35], but not whole inactivated viruses. Electrochemical impedance spectroscopy (EIS)-based aptasensors are especially attractive due to their simple sensor fabrication, rapid detection process, and high sensitivity. EIS aptasensors are a type of label-free biosensor that realizes target detection by measuring the changes in charge-transfer resistance in the presence of a redox marker upon conformational changes in the aptamer on an interaction surface [15]. Several EIS aptasensors have been analyzed for the detection of viruses, including Zika virus [36], avian flu [37], and SARS-CoV-2 [38,39] (based on aptamers against spike glycoprotein and/or nucleocapsid protein), and they were based on gold electrodes or non-gold electrodes, but no EIS aptasensors based on aptamers against inactivated SARS-CoV-2 have yet been reported.

Both FOEW and EIS are suitable sensing platforms for rapid detection, but they both possess weaknesses. For example, the sensitivity of FOEW is typically lower than that of EIS, while the whole detection time using FOEW (typically 5–6 min) is much shorter than that of EIS (typically 15–30 min). In addition, in FOEW, the aptamer is covalently functionalized on the optical fiber, rendering its high stability and long shelf-time (more than one year at room temperature) less effective. In contrast, when the gold electrode is used, the aptamer is immobilized on the gold surface via the formation of the Au-S bond. It is widely recognized that the Au-S bond is easily oxidized by air, resulting in the dissociation of the aptamer from the electrode and therefore the short shelf-time (typically less than one week exposed to the air). FOEW and EIS could be selectively used to meet the specific requirements under different application situations.

In this study, we analyzed the isolation of a highly sensitive and specific DNA aptamer that targeted inactivated SARS-CoV-2 viruses via MCP–SELEX. This then permitted us to avoid biosafety issues for viable virus detection. By using the selected aptamer, we constructed an FOEW aptasensor and an electrochemical impedance aptasensor for the detection of inactivated SARS-CoV-2 viruses. Both aptasensors exhibited excellent sensitivity and selectivity for inactivated SARS-CoV-2, providing a novel means of rapid detection of SARS-CoV-2 with high biosafety.

## 2. Materials and Methods

### 2.1. Materials

Carboxylic acid group-modified magnetic beads (Dynabeads™ M-270) and phosphate-buffered saline (PBS, pH 7.4) were bought from Thermo Fisher Scientific (Waltham, MA, USA). The DNA sequences (Supplementary Information, Table S1) were synthesized by Sangon Biotech Co., Ltd. (Shanghai, China). The inactivated SARS-CoV-2 virus was kindly provided by Sinovac Biotech Ltd. (Beijing, China). The recombinant spike protein of SARS-CoV-2 was kindly provided by Beijing Institute of Basic Medical Sciences (Beijing, China). Inactivated H1N1 was kindly provided by the State Key Laboratory of Pathogen and Biosecurity, Beijing Institute of Microbiology and Epidemiology (Beijing, China). In addition, 2-(N-morpholino) ethane sulfonic acid (MES), 1-ethyl-3-(3-dimethylaminopropyl)-1-carbodiimide hydrochloride (EDC), N-hydroxysuccinimide ester (NHS), glutaraldehyde (GA), 3-aminopropyltriethoxysilane (APTS), mercaptohexanol (MCH), sodium dodecyl sulfate (SDS), sodium borohydride (NaBH_4_), and bovine serum albumin (BSA) were purchased from Sigma-Aldrich (St. Louis, MO, USA). Tris-(2-carboxyethyl) phosphine hydrochloride (TCEP) was purchased from Shanghai Yuanye Bio-Technology Co., Ltd. (Shanghai, China). Methylbenzene, hydrogen peroxide (H_2_O_2_, 30 wt.% in water), sulfuric acid, NaCl, KCl, MgCl_2_, CaCl_2_, Na_2_CO_3_, and NaHCO_3_ were purchased from Sinopharm Chemical Reagent Co., Ltd. (Beijing, China). In addition, 4-(2-hydroxyethyl) piperazine-1-ethane-sulfonic acid (HEPES) was bought from Beijing Solarbio Science and Technology Co., Ltd. (Beijing, China). The binding buffer (pH 7.4) for SARS-CoV-2 contained 50 mM HEPES, 100 mM NaCl, 5 mM KCl, 2 mM MgCl_2_, and 1 mM CaCl_2_. All chemicals were used as received without further purification. Colloidal gold test strips for inactivated SARS-CoV-2 detection were bought from Zhejiang Orient Gene Biotech Co., Ltd. (Hangzhou, China) and Guangzhou Wondfo Biotech Co., Ltd. (Guangzhou, China). RT-PCR test kits for viral RNA tests were purchased from Wuhan Easy Diagnosis Biomedicine Co., Ltd. (Wuhan, China). Multiple-mode optical fibers (UV 576/600) with a length of 5 cm and a core diameter of 600 µm were purchased from Beijing Scitlion Technology Co., Ltd. (Beijing, China). Gold working electrodes and Al_2_O_3_ powder for electrode polishing were purchased from Shanghai Chenhua Co. Ltd. (Shanghai, China).

### 2.2. MCP–SELEX Procedure

The inactivated SARS-CoV-2 (strain CN2) was prepared by Sinovac Biotech Ltd. (Beijing, China). The active SARS-CoV-2 was collected from patients across the world and used for the generation of a vaccine. The inactivated SARS-CoV-2 was prepared using a well-established protocol [40]. Basically, the virus was chemically inactivated using β-propiolactone, and further purified using depth filtration and chromatography.

Aptamers for inactivated SARS-CoV-2 were isolated and selected by following a previously reported method [31] with modifications. The heat-denatured initial (1 nmol) or enriched (10–129 pmol) libraries of single-stranded DNAs (ssDNAs) (GP35) were incubated with 1 μg of inactivated SARS-CoV-2 in the binding buffer at room temperature for 60 min (Supplementary Information, Table S2). The inactivated SARS-CoV-2 virus was quantified with the protein concentration using a Bradford assay. Meanwhile, the magnetic beads (M-270) were activated by incubating with the EDC (50 mg/mL) and NHS (50 mg/mL) in the MES solution (25 mM, pH 5.0) for 30 min according to the NHS-assisted EDC-mediated coupling protocol (Supplementary Information, Figure S1) [41]. Specifically, the carboxylic acid groups on the magnetic beads were first transformed into the NHS ester by sequentially reacting with EDC and NHS. The NHS ester is highly reactive to the primary amino groups of the proteins expressed on the surface of the inactivated virus. The reaction between the NHS ester and the primary amino group formed the covalent amide bond. Thus, the aptamers bound with the inactivated virus were captured by the magnetic beads via the crosslinking reaction. The activated magnetic beads were incubated with a library–virus mixture for 30 min at room temperature. The beads were in excess to ensure that all viruses and library ssDNA–virus complexes were captured. After washing out unbound ssDNA through magnetic separation, the bound DNA was eluted and collected by heating at 95 °C for 20 min, and this was used as a template for the preparation of an enriched library. The enriched ssDNA library was prepared using PCR amplification, with the isolation and recycling of ssDNA from gel, by following a previously reported method [42]. After six rounds of selection, the prepared enriched pool was high-throughput sequenced, and 10 selected candidates were screened with an enzyme-linked oligonucleotide assay (ELONA) according to the detailed manipulations introduced in the following section.

### 2.3. Affinity Determination and Specificity Tests of Aptamers via ELONA

The binding affinity and specificity of the selected aptamers were characterized with ELONA. The wells were coated with inactivated SARS-CoV-2 (1 μg/well) overnight in a coating buffer (50 mM Na_2_CO_3_ and NaHCO_3_, pH 9.7). Biotin-labeled aptamers with concentrations of 0.78, 1.56, 3.125, 6.25, 12.5, 25, and 100 nM were correspondingly added into the wells. After horseradish peroxidase (HRP) conjugate incubation, the streptavidin-HRP conjugate identifies the biotin groups of aptamers, followed by the chromogenic reaction by using 3,3,5,5-tetramethylbenzidine (TMB). The absorbance of each well at 450 nm was recorded using a microplate reader (SpectraMax i3X, Molecular Devices). The dissociation constant (K_D_) was evaluated by a non-linear fitting of the relation between the absorbance at 450 nm (Y) and the aptamer concentration (X) based on the equation [30], Y = B_max_ X/(K_D_ + X), where B_max_ is the fitted maximum absorbance, using the software Origin. Inactivated H1N1 and its specific aptamer (termed “A20s”) were used as a control in the specificity test.

### 2.4. Fabrication of the FOEW Aptasensor

Optical fibers were treated and functionalized according to a reported method [32] with minor modifications. Briefly, approximately 3.5 cm of the resin layer of a fiber was removed. A tapered structure was formed by etching the fiber in HF until a tip diameter of approximately 250 µm was obtained. The fiber was further cleaned and hydroxylated in freshly prepared piranha solution (H_2_SO_4_/H_2_O_2_ = 3:1 *v*/*v*) after scraping off the remaining cladding. A silanization reaction was conducted to coat the surface with primary amines by incubating the fibers in a toluene solution containing 2% (*v*/*v*) APTS. After removing the excess solution, aldehyde groups were introduced with 2% (*v*/*v*) cross-linking reagent (GA) at room temperature for 3 h. The fibers were next covalently functionalized with aptamers by incubating them in 50 nM amino-modified DNA aptamers at room temperature for 6 h. Excess reactive sites (aldehyde groups, Schiff bases) were neutralized by incubating the fibers in 3% (*w*/*v*) NaBH_4_ for 15 min [43]. The fibers were then stored in a humid chamber at 4 °C. Prior to detection, the optical fibers were immersed in 1% (*v*/*v*) Tween 80 aqueous solution for 60 min to block non-specific binding sites.

Complementary short chains of the aptamers for the establishment of the FOEW aptasensor were optimized via ELONA. Inactivated SARS-CoV-2 (1 μg/well) was incubated in a 96-well plate overnight in a coating buffer (50 mM Na_2_CO_3_ and NaHCO_3_, pH 9.7). Biotin-labeled aptamer 6.9 (0.1 pmol) was incubated with complementary short chains (1 pmol) for 30 min at 37 °C. After the hybridization of aptamer 6.9 and the short chains, the mixture was added into the wells coated with inactivated SARS-CoV-2. The results of ELONA were evaluated based on the absorbance measured at 450 nm.

### 2.5. Detection of Inactivated SARS-CoV-2 with the FOEW Aptasensor

Inactivated SARS-CoV-2 was diluted with the binding buffer into a series of SARS-CoV-2 solutions with concentrations ranging from 2 pg/mL to 20 ng/mL. Samples were prepared by mixing the inactivated SARS-CoV-2 with fluorophore-labeled complementary DNA (Cy 5.5-cDNA) with a final concentration of 20 nM, and introduced into the reaction chamber of the FOEW equipment, which was purchased from Beijing Reliance S&T Co. Ltd. The interactions between immobilized aptamers and SARS-CoV-2 on the optical fibers lasted for 180 s, and they were followed by a regeneration process by flushing the regeneration buffer into the chamber for 60 s. For the specificity tests, different targets (H1N1, *S. aureus*, and BSA) with the same concentration (20 ng/mL) as that of SARS-CoV-2 were processed while following the same process. To investigate the practical application potential of the FOEW aptasensor, the detection of SARS-CoV-2 in throat swab samples was carried out by using a standard addition method. Throat swab samples collected from healthy people were incubated in 300 µL of 0.9% (*m*/*v*) saline. The solution was diluted 10 times prior to use.

### 2.6. Fabrication of the EIS Aptasensor

Gold working electrodes were cleaned and functionalized according to a published protocol [44]. In brief, the gold working electrodes were incubated in freshly prepared piranha solution for 15 min and polished with 0.05 μm Al_2_O_3_ powder using a piece of microcloth. Then, the electrodes were further cleaned by sequentially conducting cyclic voltammetry with 0.5 M NaOH, 0.5 M H_2_SO_4_, and 0.01 M KCl/0.1 M H_2_SO_4_. The thiolated aptamer 6.9 (0.1 μM) was incubated with TCEP in the binding buffer for 60 min to reduce the disulfide bonds. Then, the cleaned gold electrodes were incubated in this solution overnight to modify the gold surface with aptamers by forming gold–thiol bonds. Afterwards, the electrodes were rinsed with the binding buffer and incubated in aqueous 1 mM MCH solution for 1 h. The functionalized electrodes were thoroughly rinsed three times prior to use.

### 2.7. Detection of Inactivated SARS-CoV-2 with the EIS Aptasensor

An electrolyte solution was prepared by dissolving 5 mM K_3_[Fe(CN)_6_] and K_4_[Fe(CN)_6_] in the phosphate-buffered saline (1 M NaCl, 2.7 mM KCl, 1.8 mM KH_2_PO_4_, 8 mM Na_2_HPO_4_). EIS experiments were performed by superimposing an AC potential of 10 mV peak-to-peak amplitude on the open circuit potential of 0.26 V over a frequency range from 200 kHz to 0.03 Hz. Inactivated SARS-CoV-2 solutions with concentrations ranging from 100 fg/mL to 100 ng/mL were prepared in the binding buffer. The prepared working electrodes were incubated with inactivated SARS-CoV-2 solution for 10 min at room temperature. Electrochemical impedance spectra were collected with an electrochemical workstation from Shanghai Chenhua Co. Ltd. (CHI 604E), Shanghai, China. The spectra were analyzed with the installed commercial software Zview (Version 3.1c).

### 2.8. Detection of Inactivated SARS-CoV-2 with Commercial Colloidal Gold Test Strips and RT-PCR

The detection of inactivated SARS-CoV-2 with commercial colloidal gold test strips was conducted by following the instructions. Samples with inactivated SARS-CoV-2 (final concentrations of 1 ng/mL, 5 ng/mL, 10 ng/mL, 20 ng/mL, and 200 ng/mL) were prepared with lysis solution. RT-PCR for viral RNA tests was conducted following the instructions (Ct < 38, RT-PCR-positive). Inactivated SARS-CoV-2 solutions (1 fg/mL, 10 fg/mL, 20 pg/mL, 200 pg/mL, 2 ng/mL, 10 ng/mL, and 200 ng/mL) were prepared in the binding buffer.

## 3. Results and Discussion

### 3.1. Aptamer Selection for Inactivated SARS-CoV-2

**Figure 1:**
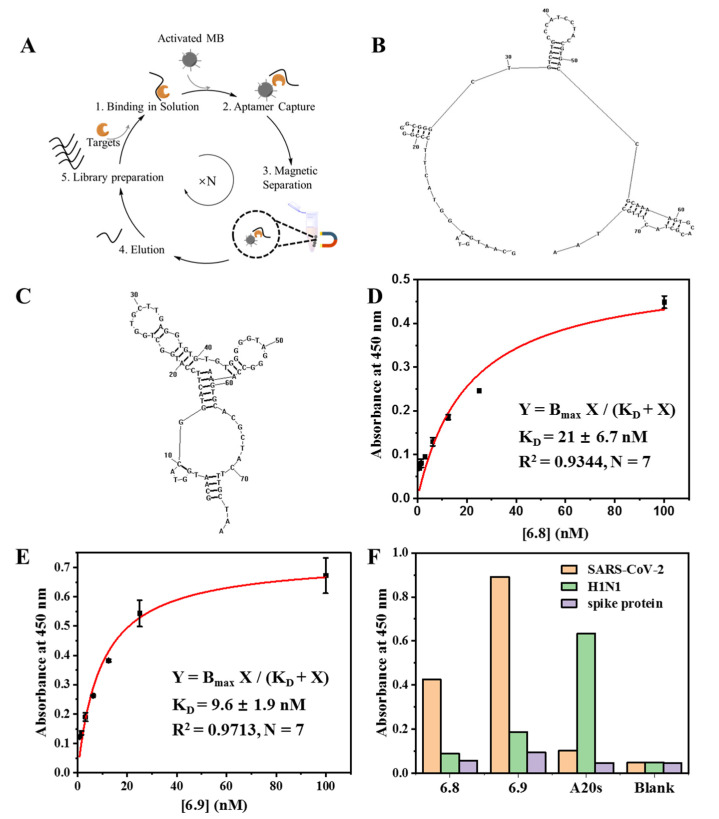
In vitro isolation of inactivated SARS-CoV-2-binding aptamers and their characterization. (**A**) Schematic illustration of MCP–SELEX for inactivated SARS-CoV-2-binding aptamer selection; the predicted secondary structure of DNA aptamer 6.8 (**B**) and aptamer 6.9 (**C**); determination of the K_D_ values of aptamer 6.8 (**D**) and aptamer 6.9 (**E**) via ELONA; (**F**) characterization of the specificity of aptamer 6.8 and aptamer 6.9 for inactivated SARS-CoV-2.

MCP–SELEX [31], which has the advantages of avoiding complicated interface interactions between immobilized targets and aptamers and ensuring highly effective isolation of aptamers, was used for the isolation of aptamers that targeted inactivated SARS-CoV-2, as illustrated in Figure 1A. Considering the biosafety issues and the convenience of future operation, the inactivated SARS-CoV-2 was used here. Inactivated SARS-CoV-2 was mixed and bound with DNA in solution, followed by aptamer isolation based on the covalent bonds between primary amino groups on the targets and the pre-activated carboxyl groups on magnetic beads. After six rounds of screening, the enriched libraries were sequenced, and ten candidate aptamers were chosen and listed in Table S3. The relative binding affinity of the candidates for inactivated SARS-CoV-2 was measured with ELONA, and the results (Supplementary Information, Figure S2) indicated that aptamer 6.8 and aptamer 6.9 showed the highest affinity. The predicted secondary structures (RNA structures) of aptamer 6.8 and aptamer 6.9 are shown in Figure 1B,C. Aptamer 6.8 showed a partially folded secondary structure, where a stem-loop structure with five base pairs and one T-G mismatch was formed in the evolved random sequence region from 31- to 52-nucleotide. The other two stem-loop structures all contained part of the primer binding sequences. The stem-loop structure in the evolved random sequence region could be the major binding site and will be further investigated in the future. Aptamer 6.9 formed a more folded and stabilized structure compared to Aptamer 6.8, which could be more favorable for binding. The K_D_ values of aptamer 6.8 and aptamer 6.9 were 21 ± 6.7 nM (Figure 1D) and 9.6 ± 1.9 nM (Figure 1E), respectively. The determination of the K_D_ values in the nanomolar range indicated the high affinities of the aptamers for SARS-CoV-2. Meanwhile, the lower K_D_ value of aptamer 6.9 demonstrated that it had a slightly higher affinity than that of aptamer 6.8 for SARS-CoV-2. The results (Figure 1F) demonstrated that our aptamers recognized the inactivated SARS-CoV-2 instead of the spike protein of SARS-CoV-2, and showed good specificity over H1N1. Aptamer 6.9 exhibited better performance against SARS-CoV-2 than that of aptamer 6.8 (Figure 1F). Thus, aptamer 6.9 was chosen for the fabrication of aptasensors for the following experiments.

### 3.2. Establishment of the FOEW Aptasensor for the Detection of Inactivated SARS-CoV-2

**Figure 2:**
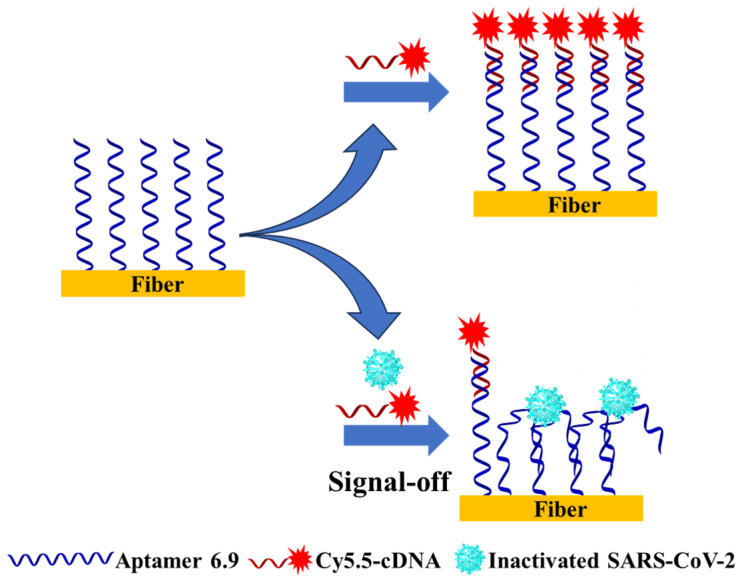
Schematic illustration of the competitive detection of SARS-CoV-2 using the FOEW aptasensor.

The effective depth of the evanescent field on the surface of an optical fiber is around 100 nm [45], and the size of a SARS-CoV-2 virus is 60–140 nm [46]. The estimated size of aptamer 6.9 or cDNA is several nanometers. There are two types of sensor designs, the aptamer- or target (SARS-CoV-2) immobilization-based sensors. In both designs, the fluorophore Cy5.5-labeled cDNA or aptamer 6.9 is utilized. If the SARS-CoV-2 is immobilized on the optical fiber, some of the Cy5.5 groups on the bound aptamer 6.9 cannot be effectively excited by the evanescent wave due to the large size of SARS-CoV-2. Therefore, to achieve a strong fluorescence excitation of a fluorophore within an evanescent field, we designed an FOEW sensor based on immobilization with aptamer 6.9 instead of a target immobilization-based sensor. As illustrated in Figure 2, we established an indirect competitive mode for the detection of inactivated SARS-CoV-2. Working samples were prepared by mixing the targets and the Cy5.5-cDNA prior to injection into the reaction chamber. On the surface of the optical fibers, the virus competed with Cy5.5-cDNA for binding with immobilized aptamers. Due to the high affinity between the targets and aptamers, immobilized aptamers would interact with the targets, losing their ability to hybridize with the cDNA. The Cy5.5-cDNA in the medium was captured by unbound aptamers on the surface and excited by evanescent waves. As the concentration of SARS-CoV-2 in the sample increased, more immobilized aptamers were occupied, leaving fewer binding sites for Cy5.5-cDNA. Therefore, the fluorescence intensity was expected to decrease with the increase in the target concentration, enabling the competitive detection of inactivated SARS-CoV-2 in the “signal-off” mode.

**Figure 3:**
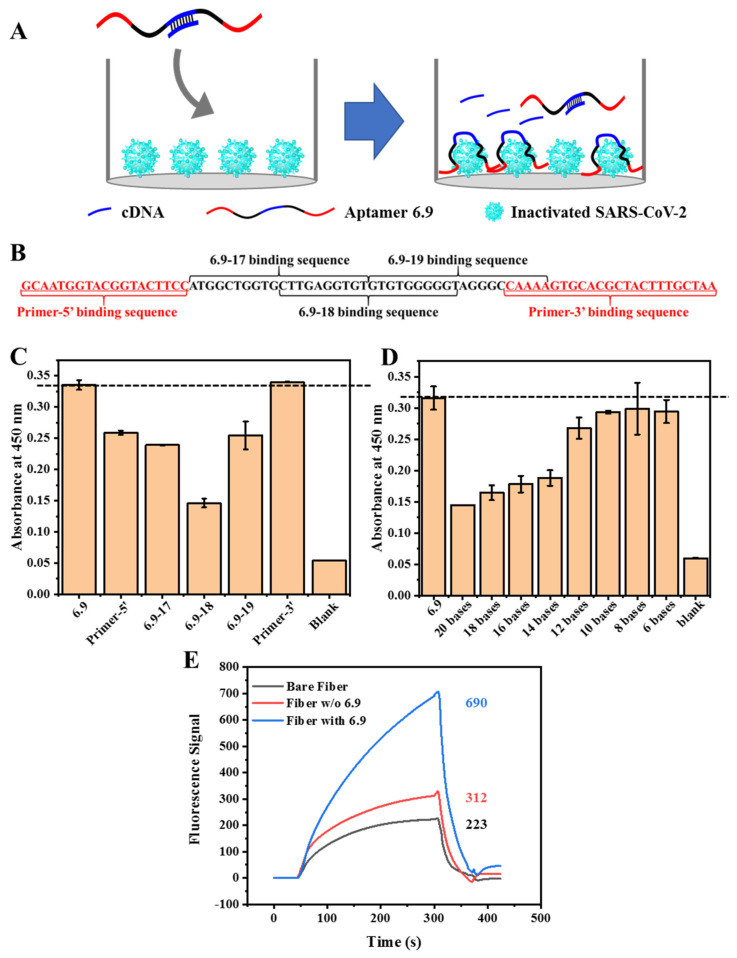
Establishment of the FOEW aptasensor. (**A**–**D**) The optimization of the cDNA used in the FOEW aptasensor. (**A**) Schematic illustration of ELONA for the characterization of cDNA optimization. (**B**) The five main regions into which aptamer 6.9 was divided; the primer binding sequence is in red. The optimization results in the hybridization locations (**C**) and the length (**D**) of cDNA used in the FOEW aptasensor for the detection of inactivated SARS-CoV-2. (**E**) The characterization of the modifications of the optical fibers by introducing 20 nM Cy5.5-cDNA in binding buffer. Black line: bare fiber; red line: fiber without aptamer; blue line: fiber functionalized with aptamer.

Cy5.5-cDNA was a crucial factor that strongly impacted the sensitivity of the FOEW sensor. We optimized the sequence and length of the cDNA used in the detection system via ELONA (Figure 3A). From the 5′ end to the 3′ end, based on the sequence and secondary structure of aptamer 6.9, we divided it into five regions, namely, the primer regions on each end and three regions with an equal length (20 bases), which were mostly in the variable region (Figure 3B). Among the five complementary DNA chains (Supplementary Information, Table S4), 6.9–18 (in the middle of the variable region), which had a 56% signal decrease, showed the greatest impact on hindering the binding between the aptamers and targets (Figure 3C). To further study the effects of the length of complementary short chains on the competitive relationship between cDNAs and targets, cDNAs (Supplementary Information, Table S4) with lengths of 6, 8, 10, 12, 14, 16, 18, and 20 -mer were designed by truncating one base from each end of 6.9–18, and they were tested separately. The results showed that cDNAs with more than 12 bases exhibited competitive abilities for aptamer–target binding (Figure 3D). However, the cDNAs that were shorter than 12 bases showed little competition with the target for aptamer binding. Thus, 6.9-18-3 with a length of 12 bases was chosen as the competitive cDNA for the construction of the FOEW aptasensor.

**Figure 4:**
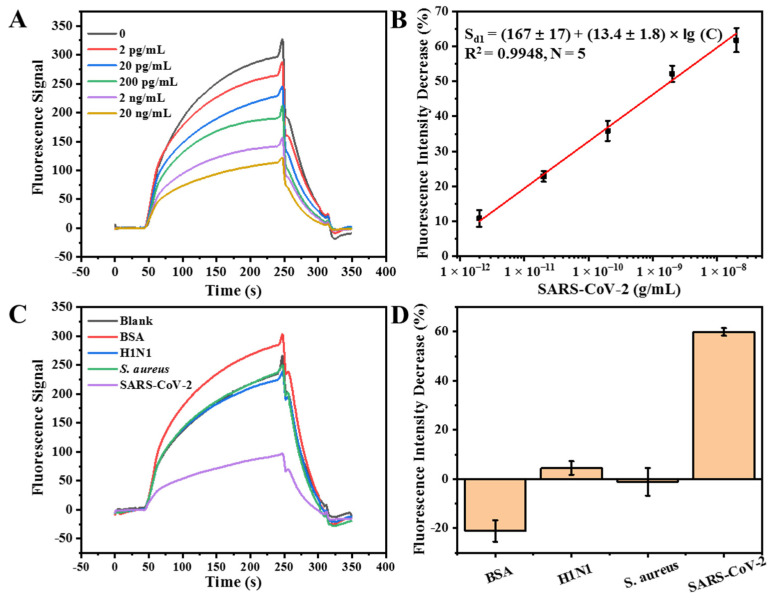
Detection of inactivated SARS-CoV-2 using the FOEW aptasensor. (**A**) The original real-time fluorescence titration signals for the establishment of calibration plot. The concentration of inactivated SARS-CoV-2 used were 2 pg/mL, 20 pg/mL, 200 pg/mL, 2 ng/mL and 20 ng/mL; (**B**) the corresponding standard calibration plot; (**C**) the real-time fluorescence signals of the specificity tests by using 20 ng/mL BSA, H1N1, *S. aureus*, and inactivated SARS-CoV-2; (**D**) the corresponding specificity results shown in terms of the relative signal decrease (%).

A standard fluorescence signal trajectory of an FOEW detection cycle is presented in Figure 4. The detection baseline value was obtained by collecting the fluorescence signal values at 25 s, while introducing the binding buffer into the reaction chamber. The sample was injected at 30–235 s, during which the fluorescence signal continuously increased and reached a plateau at the end. Afterwards, the sensor was regenerated by flowing through the regeneration buffer for 60 s. The noncovalently bound target–aptamer complexes and hybridized DNA were dissociated, resulting in a return of the fluorescence signal to the baseline. Lastly, we continued to thoroughly wash the chamber with the binding buffer for 45 s to prepare the FOEW standby for the next run. To avoid batch-to-batch differences, the baseline subtracted fluorescence signals were used (F = F_p_ − F_b_, F: baseline subtracted fluorescence; F_p_: peak value with fluorescence collected at 235 s; F_b_: baseline value with fluorescence collected at 25 s).

The characterization of the modification of the optic fibers was conducted using three main intermediates—bare fiber and fibers without and with immobilized aptamer. The bare fiber was the only fiber treated by HF etching. The fiber without aptamer was prepared the same as the fiber functionalized with aptamer except the aptamer immobilization step. The fluorescence signals of those three fibers were collected by introducing Cy5.5-cDNA into the reaction chamber. The intensity of the fiber with immobilized aptamers was more than two and three times higher than those of the fiber without aptamer and the bare fiber (Figure 3E), respectively. The bare fiber was negatively charged due to the silanol groups on the surface and, therefore, restrained the adsorption of negatively charged DNA, showing the lowest intensity. For the fiber before aptamer modification, after silanization and crosslinking functionalization, the surface was coated with aldehyde groups and a small portion of unreacted amino groups that were positively charged. The fluorescence signal slightly increased compared with that of the bare fiber, which was attributed to the surface modification. After aptamer immobilization, a dramatically increased signal was observed due to the hybridization between cDNA and immobilized aptamers. The sequentially increased fluorescence intensity confirmed the successful modification of the aptamers on the optical fibers.

### 3.3. Detection of Inactivated SARS-CoV-2 with the FOEW Aptasensor

A dose–response plot was constructed by performing sequential injections of samples containing inactivated SARS-CoV-2 at various concentrations ranging from 0 to 20 ng/mL and Cy5.5-cDNA into the chamber of the FOEW aptasensor. The fluorescence signal proportionally decreased with the increase in the target concentration (Figure 4A). To avoid batch-to-batch differences, the fluorescence signal (F) obtained was normalized with the blank signal (F_0_). The signal decrease percentage (S_d1_, S_d1_ = [(F_0_ − F)/F_0_] × 100%) was used to create the calibration plot. The semi-log linear range for SARS-CoV-2 detection, as shown in Figure 4B, was from 2 pg/mL to 20 ng/mL, and the calibration plot can be represented by the expression S_d1_ = (167 ± 17) + (13.4 ± 1.8) × lg (C) (R^2^ = 0.9948, N = 5, 95% confidence intervals with three degrees of freedom), where C denotes the concentration of SARS-CoV-2 (g/mL). The standard deviations of the triplicates were lower than 5% (Figure 4B). Based on a signal-to-noise ratio of three, the limit of detection (LOD) was estimated to be 740 fg/mL. We established a sensitive FOEW aptasensor for the detection of inactivated SARS-CoV-2.

For the selectivity test of the FOEW aptasensor for SARS-CoV-2, we selected three interferences with different structures and sizes, namely, BSA (M.W. 66.4 kDa; 140 × 40 × 40 Å), inactivated H1N1 (80–120 nm in diameter) [47], and fixed *S. aureus* (1.0–1.5 µm in diameter) [48]. BSA is a commonly used globular protein. H1N1, a type of influenza A virus, has a similar size and transmission mode to that of SARS-CoV-2. More importantly, influenza A and COVID-19 often occur in the same transmission season, which makes it of great significance to distinguish influenza A virus from SARS-CoV-2. *S. aureus* is a common pathogenic microorganism. The positive signal change was observed in the presence of BSA (Figure 4C,D) due to the BSA-enhanced non-specific adsorption of Cy5.5-labeled cDNA on the surface of the optical fiber. The weak negative signal changes were observed in the presence of inactivated H1N1 and fixed *S. aureus* (Figure 4C,D), suggesting that both of them only weakly competed with cDNA for the binding with aptamer 6.9. Therefore, the results indicated that the FOEW aptasensor that we constructed had high selectivity and practical potential.

To investigate the practical application of the FOEW aptasensor for the detection of SARS-CoV-2, it was evaluated with the throat swab samples using the standard addition method. The average recoveries of triplicate experiments with the addition of 0.5 and 5 ng/mL of SARS-CoV-2 were 98.7% and 95.7%, respectively; detailed information is shown in Table S5. There was no matrix interference from the 10-fold-diluted throat swab samples. This allowed for easy manipulation without any pretreatment. Together, the results verified that our FOEW aptasensor exhibited good potential for SARS-CoV-2 detection in biological systems.

### 3.4. Establishment of the EIS Aptasensor for the Detection of Inactivated SARS-CoV-2

**Figure 5:**
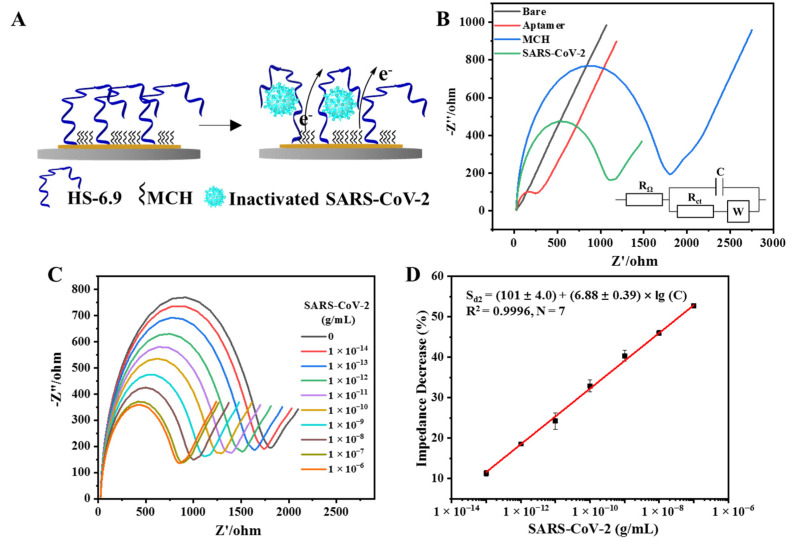
Detection of inactivated SARS-CoV-2 using the EIS aptasensor. (**A**) Schematic illustration of the EIS aptasensor for SARS-CoV-2 detection; (**B**) the characterization of the gold electrode modified with aptamer 6.9 and its response to 1 ng/mL inactivated SARS-CoV-2 in the binding buffer, with the inset figure illustrating the equivalent circuit for the fitting analysis; (**C**) the original spectra collected for inactivated SARS-CoV-2 detection in binding buffer with the concentration ranging from 10 fg/mL to 1 µg/mL; (**D**) the calibration plot for SARS-CoV-2 detection in binding buffer.

To investigate the feasibility of the selected aptamer for various techniques, we further fabricated an EIS aptasensor for the rapid detection of inactivated SARS-CoV-2, while considering the advantages of this electrochemical aptasensor in terms of ultra-sensitivity, miniaturization, and portability [49,50]. The binding between immobilized aptamers and the virus resulted in a significant decrease in the impedance, enabling the sensitive recognition of inactivated SARS-CoV-2 (Figure 5). The specific response of decreased impedance to SARS-CoV-2 should be attributed to the binding-induced folding of aptamer 6.9 into a more compact conformation (Figure 5A), leading to the higher accessibility of the electrons and the higher electron exchange rate of [Fe(CN)_6_] ^3−/4−^ on the electrode surface. In Figure 5B, a typical Nyquist (imaginary impedance (Z″) versus real impedance (Z′)) plot shows a semicircular feature in the high-frequency region and a linear impedance in the low-frequency region, which have arisen from the electron transport and diffusion process of [Fe(CN)_6_]^3−^/[Fe(CN)_6_]^4−^ in the vicinity of the electrode in 0.01 M phosphate-buffered saline (pH 7.4), respectively. The equivalent circuit that we used for the fitting analysis (Figure 5B, inset figure) included the solution resistance (R_Ω_), electron transport resistance (R_ct_), double-layer capacitance (C), and Warburg impedance (W). In each Nyquist plot, the diameter of the semicircular feature was used to estimate R_ct_ of [Fe(CN)_6_]^3−^/[Fe(CN)_6_]^4−^ at the electrode. The experimental and simulated Nyquist plots of interfacial processes for the establishment of the EIS aptasensor were shown in Figure S3, with the calculated values of all elements in Table S6.

The spectrum of the bare gold working electrode (Figure 5B, dark line) was almost a straight line, indicative of a diffusion process. After the modification with aptamers, the R_ct_ increased by five times (compared with that of the bare gold electrode, Figure 5B, red line, Table S6), which was caused by the immobilized negatively charged DNA repelling the redox marker [Fe(CN)_6_]^3−/4−^ from the electrode and reducing the electron transfer rate. The binding sites of the gold surface were further blocked by MCH, resulting in a much larger increase in impedance (around 30 times that of the bare gold electrode, Figure 5B, blue line). This was because MCH formed a closed layer on the electrode surface that could effectively isolate material exchange and electron transfer between the electrode surface and the external environment. The electrochemical impedance was decreased by 40% (compared with that of the electrode after MCH blocking) after the electrode was incubated with 1 ng/mL SARS-CoV-2 (Figure 5B, green line). The binding between the immobilized aptamers and the inactivated SARS-CoV-2 may have altered the conformation of the aptamers into more compact ones, leading to easier electron exchange and a decrease in impedance, thus indicating the successful fabrication of an EIS aptasensor for SARS-CoV-2.

### 3.5. Detection of Inactivated SARS-CoV-2 with the EIS Aptasensor

The developed EIS aptasensor was validated by detecting increasing SARS-CoV-2 concentration from 1 × 10^−14^ g/mL to 1 × 10^−6^ g/mL using EIS. All Nyquist plots obtained are shown in Figure 5C. The R_ct_ from each Nyquist plot was then measured (Supplementary Information, Table S7). The corresponding signal decrease percentage (S_d2_: signal decrease, S_d2_ = [(R_ct,0_ − R_ct_)/R_ct,0_] × 100%, R_ct,0_: R_ct_ value measured without SARS-CoV-2) was then evaluated and plotted against log10 (SARS-CoV-2 concentration; C) to obtain the calibration plot in Figure 5D, which can be represented by the expression S_d2_= (101 ± 4.0) + (6.88 ± 0.39) × lg (C), (R^2^ = 0.9996, N = 7, 95% confidence intervals with 5 degrees of freedom) with a low limit of detection of 5.1 fg/mL. We improved the sensitivity of the aptasensor on the electrochemical platform by around 100 times in comparison with that of the FOEW aptasensor.

**Figure 6:**
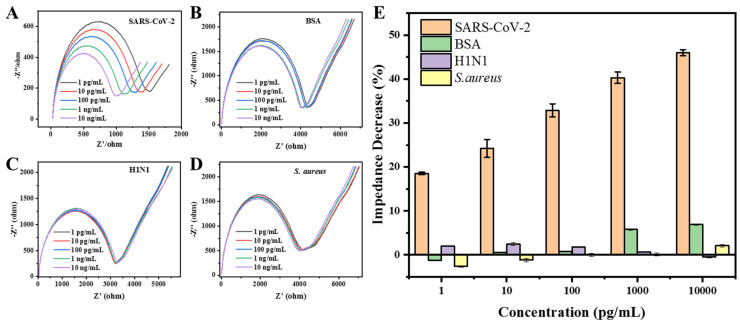
Specificity test of the EIS aptasensor for the detection of inactivated SARS-CoV-2. (**A**–**D**) The original EIS spectra collected for the detection of inactivated SARS-CoV-2, BSA, inactivated H1N1, and *S. aureus* bacteria in the binding buffer with their concentration ranging from 1 pg/mL to 10 ng/mL; (**E**) the corresponding specificity results shown in terms of the relative impedance decrease (%).

The specific response of the decreased R_ct_ to SARS-CoV-2 was verified by introducing other compositions. The R_ct_ monotonically decreased as the concentration of SARS-CoV-2 from increased 1 pg/mL to 10 ng/mL (Figure 6A). In sharp contrast, the minimal changes when introducing the inactivated H1N1 virus, the inactivated *S. aureus* bacteria, or BSA in the same concentration range (Figure 6B–D). The maximum R_ct_ decrease percentage (6.0%) was observed when the sensor was triggered by the 10 ng/mL of BSA. It was about 7.5-fold lower than that caused by SARS-CoV-2. Thus, the results illustrated the excellent specificity of our EIS aptasensor, arising from the interaction between the aptamer and SARS-CoV-2. Specifically, to further investigate the practical application of the EIS aptasensor, the average recoveries of 2 pg/mL and 20 pg/mL of SARS-CoV-2 in throat swab samples were found to be 90.7% and 88.7%, respectively. The detailed data from the triplicate experiments are shown in Table S8, and the good reliability of our established EIS aptasensor was verified.

### 3.6. Performance Evaluation

**Figure 7:**
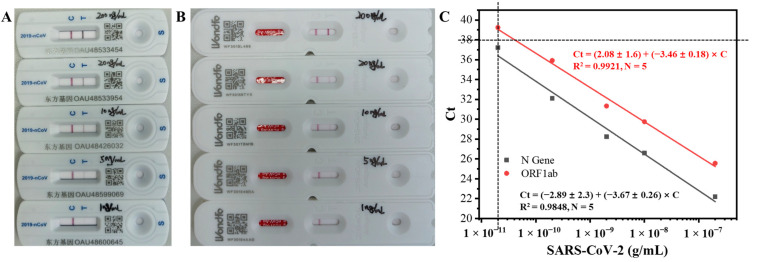
Commercial colloidal gold methods for the detection of inactivated SARS-CoV-2 using test strips from two different brands (**A**,**B**). The LODs of the inactivated SARS-CoV-2 samples were 1–5 ng/mL (**A**) and 5–10 ng/mL (**B**). (**C**) The calibration plots between the concentrations of inactivated SARS-CoV-2 and the corresponding Ct values measured with RT-PCR for the two genes (N Gene and ORF1ab).

Further, we compared the performance of our aptasensors with that of the commercial colloidal gold test strips (Figure 7A,B) and viral RNA tests with RT-PCR (Figure 7C). The detection of inactivated SARS-CoV-2 is based on the formation of the sandwich of “antibody- SARS-CoV-2 -gold nanoparticle-labeled antibody” on the “T” line for test strips. When the concentration of inactivated SARS-CoV-2 reaches a certain value, the “T” line shows a red color, suggesting a positive result. The red color of the “T” line becomes stronger as SARS-CoV-2 concentration increases. The test strips from two different brands were tested. The red “T” line, a positive response, was observed by the naked eyes when the concentration of inactivated SARS-CoV-2 was higher than 5 and 10 ng/mL for the strips from Zhejiang Orient Gene Biotech Co., Ltd. (Figure 7A) and Guangzhou Wondfo Biotech Co., Ltd. (Figure 7B), respectively. The estimated LODs for the two test strips were 1–5 and 5–10 ng/mL, respectively. Although the commercial colloidal gold test strips exhibit the benefit of easy manipulation, insensitivity is the undeniable drawback. Our aptasensors showed remarkable lower LODs, with the FOEW aptasensor (LOD = 740 fg/mL) and EIS aptasensor (LOD = 5.1 fg/mL) showing a more than 1000 and 100,000 times lower limit of detection than those of the commercial colloidal test strips (estimated LOD higher than 1 ng/mL).

RT-PCR is the gold standard method for the detection of SARS-CoV-2, where the two genome sequences, N Gene and ORFlab, are used as the target templates in RT-PCR. Typically, a positive response is given when the two Ct values are less than 38. According to the two calibration curves shown in Figure 7C, the samples were positive (Ct < 38) when the concentration of inactivated SARS-CoV-2 was higher than 20 pg/mL. Our aptasensors both exhibited the ability to distinguish RT-PCR-positive and -negative samples. The outstanding performance of the FOEW and EIS aptasensors was attributed to the binding of one virus with multiple aptamers. We established rapid and sensitive aptasensors for the detection of SARS-CoV-2.

## 4. Conclusions

Here, we analyzed an aptamer with high affinity (K_D_ of 9.6 nM) and specificity for the inactivated SARS-CoV-2 virus for the first time to avoid the issue of biosafety in the detection of viable viruses. FOEW and EIS aptasensors were developed for the detection of inactivated SARS-CoV-2. A linear detection range from 2 pg/mL to 20 ng/mL with an LOD of 740 fg/mL was obtained with the FOEW aptasensor within a six minute read-out time, thus meeting the detection requirements. The EIS aptasensor was established by anchoring aptamers on a gold electrode, resulting in a linear detection range from 100 fg/mL to 100 ng/mL and an LOD of 5.1 fg/mL. Our FOEW and EIS aptasensors were at least 1000-fold more sensitive than commercial colloidal gold test strips for the detection of the inactivated SARS-CoV-2 virus and exhibited the ability to distinguish RT-PCR positive samples. The aptasensors that we established, which have improved biosafety, allow for the realization of rapid and sensitive SARS-CoV-2 detection, and act as alternative techniques for RT-PCR for the quick identification of viral infections.

## Data Availability

The data presented in this study are available from the corresponding author upon reasonable request.

## Supplementary Materials

The following supporting information can be downloaded at: https://www.mdpi.com/article/10.3390/bios14050231/s1, Table S1: The sequences of DNA used in this research; Table S2: The inputs of libraries and inactivated SARS-CoV-2 in each round of MCP–SELEX; Table S3: The sequences of the 10 candidate aptamers; Table S4: The complementary short chains used for the optimization of the FOEW aptasensor; Table S5: Determination of the spiked SARS-CoV-2 recovery rate using the FOEW aptasensor; Table S6: Calculated values of elements in the equivalent circuit (Figure 5B) in the following interfacial processes for the establishment of the EIS aptasensor; Table S7: Calculated values of elements in the equivalent circuit (Figure 5C) for the establishment of the calibration plot; Table S8: Determination of the spiked SARS-CoV-2 recovery rate using the EIS aptasensor; Figure S1: The EDC/NHS mediated cross-linking reaction between carboxylic acid-functionalized magnetic beads and the inactivated SARSCoV-2 viruses; Figure S2: Binding affinity and specificity screening of the 10 candidate aptamers against inactivated SARS-CoV-2 with ELONA; Figure S3: The experimental (lines) and simulated (dot lines) Nyquist plots of interfacial processes for the establishment of the EIS aptasensor (modified with aptamer 6.9 and its response to 1 ng/mL inactivated SARS-CoV-2).

## References

[1] Pekosz A., Parvu V., Li M., Andrews J.C., Manabe Y.C., Kodsi S., Gary D.S., Roger-Dalbert C., Leitch J., Cooper C.K. (2021). Antigen-Based Testing but Not Real-Time Polymerase Chain Reaction Correlates with Severe Acute Respiratory Syndrome Coronavirus 2 Viral Culture. Clin. Infect. Dis..

[2] Rahbari R., Moradi N., Abdi M. (2021). RRT-PCR for SARS-CoV-2: Analytical Considerations. Clin. Chim. Acta.

[3] Araujo W.R., Lukas H., Torres M.D.T., Gao W., de la Fuente-Nunez C. (2024). Low-Cost Biosensor Technologies for Rapid Detection of COVID-19 and Future Pandemics. ACS Nano.

[4] Liu Y., Li Y., Hang Y., Wang L., Wang J., Bao N., Kim Y., Jang H.W. (2024). Rapid Assays of SARS-CoV-2 Virus and Noble Biosensors by Nanomaterials. Nano Converg..

[5] Serrano M.M., Rodríguez D.N., Palop N.T., Arenas R.O., Córdoba M.M., Mochón M.D.O., Cardona C.G. (2020). Comparison of Commercial Lateral Flow Immunoassays and ELISA for SARS-CoV-2 Antibody Detection. J. Clin. Virol..

[6] Cubas-Atienzar A.I., Kontogianni K., Edwards T., Wooding D., Buist K., Thompson C.R., Williams C.T., Patterson E.I., Hughes G.L., Baldwin L. (2021). Limit of Detection in Different Matrices of 19 Commercially Available Rapid Antigen Tests for the Detection of SARS-CoV-2. Sci. Rep..

[7] Ellington A.D., Szostak J.W. (1990). In Vitro Selection of RNA Molecules That Bind Specific Ligands. Nature.

[8] Tuerk C., Gold L. (1990). Systematic Evolution of Ligands by Exponential Enrichment: RNA Ligands to Bacteriophage T4 DNA Polymerase. Science.

[9] Qu H., Csordas A.T., Wang J., Oh S.S., Eisenstein M.S., Soh H.T. (2016). Rapid and Label-Free Strategy to Isolate Aptamers for Metal Ions. ACS Nano.

[10] Yang K., Mitchell N.M., Banerjee S., Cheng Z., Taylor S., Kostic A.M., Wong I., Sajjath S., Zhang Y., Stevens J. (2023). A Functional Group–Guided Approach to Aptamers for Small Molecules. Science.

[11] Singh N.K., Wang Y., Wen C., Davis B., Wang X., Lee K., Wang Y. (2023). High-Affinity One-Step Aptamer Selection Using a Non-Fouling Porous Hydrogel. Nat. Biotechnol..

[12] Li S., Xu H., Ding H., Huang Y., Cao X., Yang G., Li J., Xie Z., Meng Y., Li X. (2009). Identification of an Aptamer Targeting HnRNP A1 by Tissue Slide-Based SELEX. J. Pathol..

[13] Li L., Wan J., Wen X., Guo Q., Jiang H., Wang J., Ren Y., Wang K. (2021). Identification of a New DNA Aptamer by Tissue-SELEX for Cancer Recognition and Imaging. Anal. Chem..

[14] Huang L., Tian S., Zhao W., Liu K., Ma X., Guo J. (2021). Aptamer-Based Lateral Flow Assay on-Site Biosensors. Biosens. Bioelectron..

[15] Yang L.F., Ling M., Kacherovsky N., Pun S.H. (2023). Aptamers 101: Aptamer Discovery and in Vitro Applications in Biosensors and Separations. Chem. Sci..

[16] Wandtke T., Wędrowska E., Szczur M., Przybylski G., Libura M., Kopiński P. (2022). Aptamers-Diagnostic and Therapeutic Solution in SARS-CoV-2. Int. J. Mol. Sci..

[17] Lasserre P., Balansethupathy B., Vezza V.J., Butterworth A., Macdonald A., Blair E.O., McAteer L., Hannah S., Ward A.C., Hoskisson P.A. (2022). SARS-CoV-2 Aptasensors Based on Electrochemical Impedance Spectroscopy and Low-Cost Gold Electrode Substrates. Anal. Chem..

[18] Yang L.F., Kacherovsky N., Liang J., Salipante S.J., Pun S.H. (2022). SCORe: SARS-CoV-2 Omicron Variant RBD-Binding DNA Aptamer for Multiplexed Rapid Detection and Pseudovirus Neutralization. Anal. Chem..

[19] Aithal S., Mishriki S., Gupta R., Sahu R.P., Botos G. (2022). SARS-CoV-2 Detection with Aptamer-Functionalized Gold Nanoparticles. Talanta.

[20] Chauhan N., Xiong Y., Ren S., Dwivedy A., Magazine N., Zhou L., Jin X., Zhang T., Cunningham B.T., Yao S. (2023). Net-Shaped DNA Nanostructures Designed for Rapid/Sensitive Detection and Potential Inhibition of the SARS-CoV-2 Virus. J. Am. Chem. Soc..

[21] Song Y., Song J., Wei X., Huang M., Sun M., Zhu L., Lin B., Shen H., Zhu Z., Yang C. (2020). Discovery of aptamers targeting the receptor-binding domain of the SARS-CoV-2 spike glycoprotein. Anal. Chem..

[22] Cennamo N., Pasquardini L., Arcadio F., Lunelli L., Vanzetti L., Carafa V., Altucci L., Zeni L. (2021). SARS-CoV-2 spike protein detection through a plasmonic d-shaped plastic optical fiber aptasensor. Talanta.

[23] Lewis T., Giroux E., Jovic M., Martic-Milne S. (2021). Localized surface plasmon resonance aptasensor for selective detection of SARS-CoV-2 s1 protein. Analyst.

[24] Han C., Li W., Li Q., Xing W., Luo H., Ji H., Fang X., Luo Z., Zhang L. (2022). CRISPR/Cas12a-Derived Electrochemical Aptasensor for Ultrasensitive Detection of COVID-19 Nucleocapsid Protein. Biosens. Bioelectron..

[25] Zhang L., Fang X., Liu X., Ou H., Zhang H., Wang J., Li Q., Cheng H., Zhang W., Luo Z. (2020). Discovery of Sandwich Type COVID-19 Nucleocapsid Protein DNA Aptamers. Chem. Commun..

[26] Liu J., Mao J., Hou M., Hu Z., Sun G., Zhang S. (2022). A Rapid SARS-CoV-2 Nucleocapsid Protein Profiling Assay with High Sensitivity Comparable to Nucleic Acid Detection. Anal. Chem..

[27] Tian J., Liang Z., Hu O., He Q., Sun D., Chen Z. (2021). An electrochemical dual-aptamer biosensor based on metal-organic frameworksmil-53 decorated with au@pt nanoparticles and enzymes for detection of COVID-19 nucleocapsid protein. Electrochim. Acta.

[28] Ramanathan S., Gopinath S.C.B., Ismail Z.H., Md Arshad M.K., Poopalan P. (2022). Aptasensing nucleocapsid protein on nanodiamond assembled gold interdigitated electrodes for impedimetric SARS-CoV-2 infectious disease assessment. Biosens. Bioelectron..

[29] Chang T.-C., Sun A.Y., Huang Y.-C., Wang C.-H., Wang S.-C., Chau L.-K. (2022). Integration of Power-Free and Self-Contained Microfluidic Chip with Fiber Optic Particle Plasmon Resonance Aptasensor for Rapid Detection of SARS-CoV-2 Nucleocapsid Protein. Biosensors.

[30] Bai C., Lu Z., Jiang H., Yang Z., Liu X., Ding H., Li H., Dong J., Huang A., Fang T. (2018). Aptamer Selection and Application in Multivalent Binding-Based Electrical Impedance Detection of Inactivated H1N1 Virus. Biosens. Bioelectron..

[31] Qiao N., Li J., Wu X., Diao D., Zhao J., Li J., Ren X., Ding X., Shangguan D., Lou X. (2019). Speeding up in Vitro Discovery of Structure-Switching Aptamers via Magnetic Cross-Linking Precipitation. Anal. Chem..

[32] Zhou J., Li H., Li J., Liu X., Zhao J., Wang N., Wang Y., Zhang Y., Zhang X., Xin Y. (2023). Selection of Regioselective DNA Aptamer for Detection of Homocysteine in Nondeproteinized Human Plasma. Biosens. Bioelectron..

[33] Yang Y., Zhao R., Wang Y., Song D., Jiang B., Guo X., Liu W., Long F., Song H., Hao R. (2022). Rapid and Universal Detection of SARS-CoV-2 and Influenza A Virus Using a Reusable Dual-Channel Optic Fiber Immunosensor. J. Med. Virol..

[34] Long F., Zhu A., Wang H. (2014). Optofluidics-Based DNA Structure-Competitive Aptasensor for Rapid on-Site Detection of Lead(II) in an Aquatic Environment. Anal. Chim. Acta.

[35] Li S., Zhu L., Zhu L., Mei X., Xu W. (2022). A Sandwich-Based Evanescent Wave Fluorescent Biosensor for Simple, Real-Time Exosome Detection. Biosens. Bioelectron..

[36] Dolai S., Tabib-Azar M. (2020). Whole Virus Detection Using Aptamers and Paper-based Sensor Potentiometry. Med. Devices Sens..

[37] Kwon J., Lee Y., Lee T., Ahn J.H. (2020). Aptamer-Based Field-Effect Transistor for Detection of Avian Influenza Virus in Chicken Serum. Anal. Chem..

[38] Kurmangali A., Dukenbayev K., Kanayeva D. (2022). Sensitive Detection of SARS-CoV-2 Variants Using an Electrochemical Impedance Spectroscopy Based Aptasensor. Int. J. Mol. Sci..

[39] Ban D.K., Bodily T., Karkisaval A.G., Dong Y., Natani S., Ramanathan A., Ramil A., Srivastava S., Bandaru P., Glinsky G. (2022). Rapid Self-Test of Unprocessed Viruses of SARS-CoV-2 and Its Variants in Saliva by Portable Wireless Graphene Biosensor. Proc. Natl. Acad. Sci. USA.

[40] Gao Q., Bao L., Mao H., Wang L., Xu K., Yang M., Li Y., Zhu L., Wang N., Lv Z. (2020). Development of an Inactivated Vaccine Candidate for SARS-CoV-2. Science.

[41] Kaldéus T., Nordenström M., Carlmark A., Wågberg L., Malmström E. (2018). Insights into the EDC-Mediated PEGylation of Cellulose Nanofibrils and Their Colloidal Stability. Carbohydr. Polym..

[42] Cao X., Li S., Chen L., Ding H., Xu H., Huang Y., Li J., Liu N., Cao W., Zhu Y. (2009). Combining Use of a Panel of SsDNA Aptamers in the Detection of Staphylococcus Aureus. Nucleic Acids Res..

[43] Razumovitch J., De França K., Kehl F., Wiki M., Meier W., Vebert C. (2009). Optimal Hybridization Efficiency upon Immobilization of Oligonucleotide Double Helices. J. Phys. Chem. B.

[44] Xiao Y., Lai R.Y., Plaxco K.W. (2007). Preparation of Electrode-Immobilized, Redox-Modified Oligonucleotides for Electrochemical DNA and Aptamer-Based Sensing. Nat. Protoc..

[45] Ahmad M., Hench L.L. (2005). Effect of Taper Geometries and Launch Angle on Evanescent Wave Penetration Depth in Optical Fibers. Biosens. Bioelectron..

[46] Zhu N., Zhang D., Wang W., Li X., Yang B., Song J., Zhao X., Huang B., Shi W., Lu R. (2020). A Novel Coronavirus from Patients with Pneumonia in China, 2019. N. Engl. J. Med..

[47] Horiguchi Y., Goda T., Matsumoto A., Takeuchi H., Yamaoka S., Miyahara Y. (2019). Gold Nanoparticles with Ligand/Zwitterion Hybrid Layer for Individual Counting of Influenza A H1N1 Subtype Using Resistive Pulse Sensing. Langmuir.

[48] Bentley K.L.D.M., Trombetta R., Nishitani K., Bello-irizarry S.N., Ninomiya M., Zhang L., Chung H.L., Mcgrath J.L., Daiss J.L., Awad H.A. (2018). Evidence of Staphylococcus Aureus Deformation, Proliferation and Migration in Canaliculi of Live Cortical Bone in Murine Models of Osteomyelitis. J. Bone Miner. Res..

[49] Panicker L.R., Kummari S., Keerthanaa M.R., Rao Bommi J., Koteshwara Reddy K., Yugender Goud K. (2024). Trends and Challenges in Electroanalytical Biosensing Methodologies for Infectious Viral Diseases. Bioelectrochemistry.

[50] Antiochia R. (2022). Electrochemical Biosensors for SARS-CoV-2 Detection: Voltametric or Impedimetric Transduction?. Bioelectrochemistry.

