# Supplementary material for: Rapid and Sensitive Detection of Inactivated SARS-CoV-2 Virus via Fiber-Optic and Electrochemical Impedance Spectroscopy Based Aptasensors
Source: Biosensors (Basel). 2024 May 7;14(5):231. doi: 10.3390/bios14050231 (PMC11117632; doi:10.3390/bios14050231)
Supplement: Supplementary file 1 [file biosensors-14-00231-s001.zip › biosensors-2952985-supplementary.pdf]

biosensors

Supplementary Information

Rapid and Sensitive Detection of Inactivated SARS-CoV-2 Virus via Fiber-Optic and Electrochemical Impedance Spectroscopy Based Aptasensors

Can Xiao [1,†], Nan Wang [2,†], Yuechao Zhao [1,†], Xuemei Liu [1], Hui Li [1], Aixue Huang [1], Lin Wang [1], Xinhui Lou [2], Bo Gao [1,*] and Ningsheng Shao [1,*]

[1] Beijing Institute of Basic Medical Sciences, Beijing 100850, China; xiaocan@bmi.ac.cn (C.X.); zhaoyuechao@bmi.ac.cn (Y.Z.); liuxuemei@bmi.ac.cn (X.L.); lihui1@bmi.ac.cn (H.L.); huangaixue@bmi.ac.cn (A.H.); wanglin1@bmi.ac.cn (L.W.); gaobo1@bmi.ac.cn (B.G.); shaoningsheng@bmi.ac.cn (N.S.)

[2] Department of Chemistry, Capital Normal University, Xisanhuan North Road. 105, Beijing 100048, China; 2210702032@cnu.edu.cn (N.W.); xinhuilou@cnu.edu.cn (X.L.)

* Correspondence: gaobo1@bmi.ac.cn (B.G.); shaoningsheng@bmi.ac.cn (N.S.)

† These authors contributed equally to this work.

Table S1. The sequences of DNA used in this research.

Name	Sequence (5'-3')	Description
GP35	GCAATGGTACGGTACTTCC-N35-CAAAAGTGCACGCTACTT TGCTAA	Initial library used in MCP-SELEX for aptamer selection
5N10-6.9	NH_2-AAAAAAAAAA- GCAATGGTACGGTACTTCCATGGCTGGTGCTTGAGGTGTG TGTGGGGGTAGGGCCAAAAGTGCACGCTACTTTGCTAA	Amino group and spacer modified aptamer 6.9 for FOEW aptasensor
Cy5.5-6.9-18-3	Cy5.5- CCACACACACCT	Cy5.5 modified complementary short chain used in FOEW aptasensor
HS-6.9	SH-3(iSp18)- GCAATGGTACGGTACTTCCATGGCTGGTGCTTGAGGTGTG TGTGGGGGTAGGGCCAAAAGTGCACGCTACTTTGCTAA	Thiolated aptamer 6.9 for EIS aptasensor
A20s	GGACCAGTTGTCTTTCGGTCTCTACCCCAGCCCGT	Specific aptamer against inactivated H1N1

Table S2. The inputs of libraries and inactivated SARS-CoV-2 in each round of MCP–SELEX.

Round	Input library (pmol)	Input inactivated SARS-CoV-2 (μg)
1st	1000	1
2nd	129	1
3rd	62	1
4th	50	1
5th	52	1
6th	10	1

Table S3. The sequences of the 10 candidate aptamers.

Name of candidate aptamers	Sequence (5’-3’)
6.1	GCAATGGTACGGTACTTCCCTGGATGGCGTTGTCTGTGTGTTTGCTGATCAA AAGTGCACGCTACTTTGCTAA
6.2	GCAATGGTACGGTACTTCCTATGGCGATGTGTTGGCTGTGTGTGGGGTGCAA AAGTGCACGCTACTTTGCTAA
6.3	GCAATGGTACGGTACTTCCGGTCTGGTTAGGTGTTGGGCATGGTGGTTGCTT TCCAAAAGTGCACGCTACTTTGCTAA
6.4	GCAATGGTACGGTACTTCCCACCTTGGCCTCCAAAAGTGCACGCTACTTTGC TAA
6.5	GCAATGGTACGGTACTTCCACGATGGTGAGGGCTGTGTTGTTACCAAAAGTG CACGCTACTTTGCTAA
6.6	GCAATGGTACGGTACTTCCCTCCGGAATTATCTGATGCCCGCTCCAAAAGTG CACGCTACTTTGCTAA
6.7	GCAATGGTACGGTACTTCCTGTGGCGAGGTAGGTGGGGTGTGTGTGTATCCA AAAGTGCACGCTACTTTGCTAA
6.8	GCAATGGTACGGTACTTCCCGGGGCGGGCTGTCATGCCCATCCTACCGTGAC CGCAAAAGTGCACGCTACTTTGCTAA
6.9	GCAATGGTACGGTACTTCCATGGCTGGTGCTTGAGGTGTGTGTGGGGGTAGG GCCAAAAGTGCACGCTACTTTGCTAA
6.10	CAATGGTACGGTACTTCCGGAGGGACTTGGAACGGTTGTAGGTGGTTCCAAA AGTGCACGCTACTTTGCTAA

Table S4. The complementary short chains used for the optimization of the FOEW aptasensor.

Name	Length	Sequence (5’-3’)
6.9-17	20 bases	ACACCTCAAGCACCAGCCAT
6.9-18	20 bases	ACCCCCACACACACCTCAAG
6.9-19	20 bases	TTTTGGCCCTACCCCCACAC
6.9-18-1	18 bases	CCCCCACACACACCTCAA
6.9-15	16 bases	CCCCACACACACCTCA
6.9-18-2	14 bases	CCCACACACACCTC
6.9-18-3	12 bases	CCACACACACCT
6.9-18-4	10 bases	CACACACACC
6.9-18-5	8 bases	ACACACAC
6.9-18-6	6 bases	CACACA

Table S5. Determination of the spiked SARS-CoV-2 recovery rate by using the FOEW aptasensor.

Spiked inactivated SARS-CoV-2 (ng/mL)	Found (ng/mL)	Recovery (%)	Average Recovery (%)
	0.48	95	
0.5	0.51	102	98.7
	0.49	99	
	4.40	88	
5	5.43	109	95.7
	4.47	90	

Table S6. Calculated values of elements in the equivalent circuit (Figure 5B) in the following interfacial processes for the establishment of the EIS aptasensor.

Processes	R_{Ω} (Ω)	R_{ct} (kΩ)	C (μF)	W (kΩ·$s^{1/2}$)
Bare gold electrode	23	0.053	85	-
modification of aptamers	23	0.27	18	3.3
Blocking with MCH	24	1.6	0.58	7.9
SARS-CoV-2 (1 ng/mL) incubation	25	0.98	0.67	7.6

Table S7. Calculated values of elements in the equivalent circuit (Figure 5C) for the establishment of the calibration plot.

SARS-CoV-2 (g/mL)	R_{Ω} (Ω)	R_{ct} (kΩ)	C (μF)	W (kΩ·$s^{1/2}$)
0	24	1.6	0.58	7.9
1×10^{-14}	24	1.5	0.58	8.7
1×10^{-13}	24	1.4	0.58	8.5
1×10^{-12}	25	1.3	0.60	8.4
1×10^{-11}	25	1.2	0.61	7.4
1×10^{-10}	25	1.1	0.63	7.4
1×10^{-9}	25	0.98	0.67	7.6
1×10^{-8}	25	0.88	0.70	5.1
1×10^{-7}	25	0.77	0.69	4.8
1×10^{-6}	25	0.76	0.71	4.3

Table S8. Determination of the spiked SARS-CoV-2 recovery rate by using the EIS aptasensor.

Spiked inactivated SARS-CoV-2 (pg/mL)	Found (pg/mL)	Recovery (%)	Average Recovery (%)
	1.90	95	
2	1.48	74	90.7
	2.06	103	
	1.65	82	
20	1.83	91	88.7
	1.85	93	

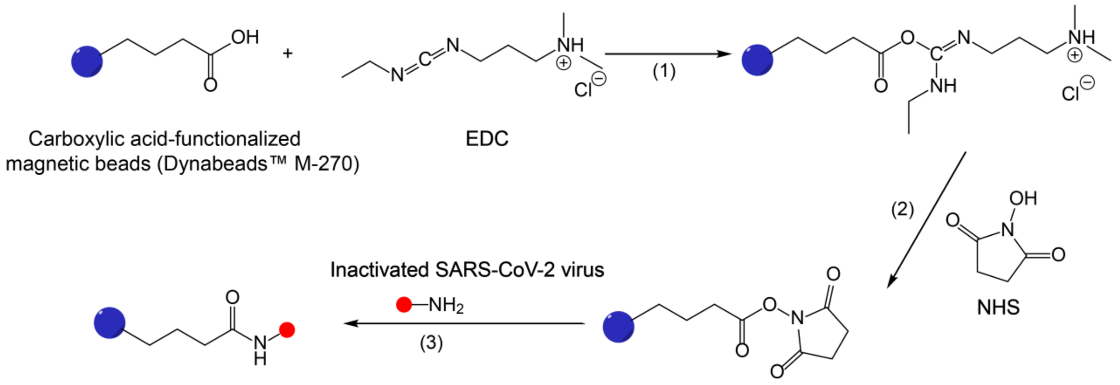

Figure S1. The EDC/NHS mediated crosslinking reaction between carboxylic acid-functionalized magnetic beads and the inactivated SARS-CoV-2 viruses.

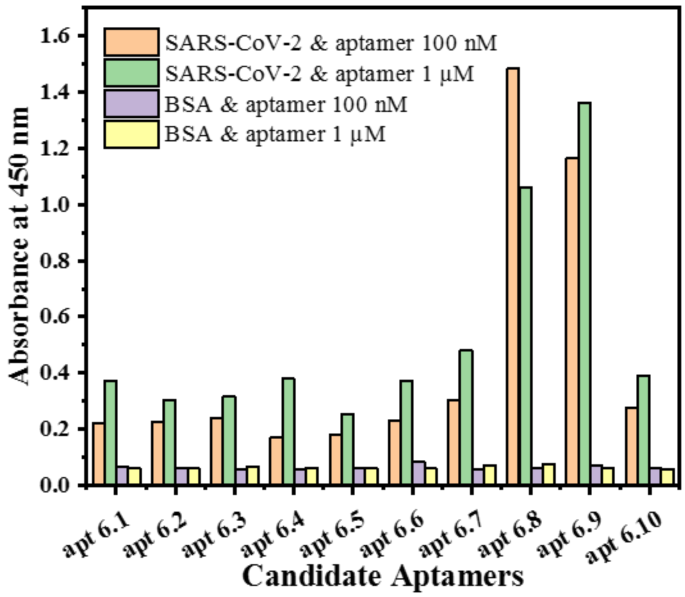

Figure S2. Binding affinity and specificity screening of the 10 candidate aptamers against inactivated SARS-CoV-2 with ELONA.

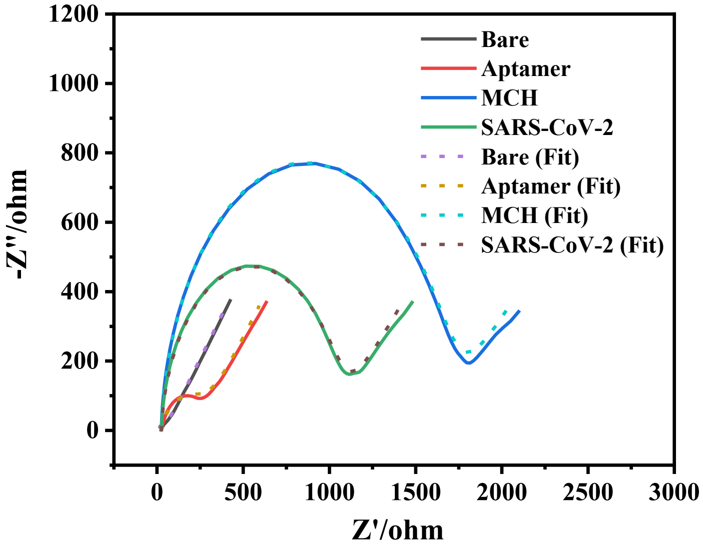

Figure S3. The experimental (lines) and simulated (dot lines) Nyquist plots of interfacial processes for the establishment of the EIS aptasensor (modified with aptamer 6.9 and its response to 1 ng/mL inactivated SARS-CoV-2).
